# Trimethylamine N-Oxide Reduces the Susceptibility of *Escherichia coli* to Multiple Antibiotics

**DOI:** 10.3389/fmicb.2022.956673

**Published:** 2022-07-07

**Authors:** Jiaxin Qiao, Yan Liang, Yao Wang, Morigen

**Affiliations:** ^1^State Key Laboratory of Reproductive Regulation and Breeding of Grassland Livestock, School of Life Sciences, Inner Mongolia University, Hohhot, China; ^2^College of Life Sciences, Inner Mongolia Agricultural University, Hohhot, China

**Keywords:** trimethylamine N-oxide, antibiotics, urea stress, disinfectants, protein denaturation, cell death, intestinal flora metabolite, *Escherichia coli*

## Abstract

Trimethylamine N-oxide (TMAO), an important intestinal flora-derived metabolite, plays a role in the development of cardiovascular disease and tumor immunity. Here, we determined the minimum inhibitory concentration (MIC) of antibiotics against *Escherichia coli* under gradient concentrations of TMAO and performed a bacterial killing analysis. Overall, TMAO (in the range of 10 ~ 100 mM) increased the MIC of quinolones, aminoglycosides, and β-lactams in a concentration-dependent manner, and increased the lethal dose of antibiotics against *E. coli*. It implies that TMAO is a potential risk for failure of anti-infective therapy, and presents a case for the relationship between intestinal flora-derived metabolites and antibiotic resistance. Further data demonstrated that the inhibition of antibiotic efficacy by TMAO is independent of the downstream metabolic processes of TMAO and the typical bacterial resistance mechanisms (*mar* motif and efflux pump). Interestingly, TMAO protects *E. coli* from high-protein denaturant (urea) stress and improves the viability of bacteria following treatment with two disinfectants (ethanol and hydrogen peroxide) that mediate protein denaturation by chemical action or oxidation. Since antibiotics can induce protein inactivation directly or indirectly, our work suggests that disruption of protein homeostasis may be a common pathway for different stress-mediated bacterial growth inhibition/cell death. In addition, we further discuss this possibility, which provides a different perspective to address the global public health problem of antibiotic resistance.

## Introduction

Trimethylamine N-oxide (TMAO) is a bioactive molecule produced by gut microbial-derived metabolism (Ufnal et al., 2015; Subramaniam and Fletcher, 2018; Gessner et al., 2020). In humans, diets rich in TMA precursors (e.g., choline and L-carnitine) are the main source of TMAO (Zhang et al., 1999; Wang et al., 2011; Koeth et al., 2013). The intestinal flora converts dietary nutrients to TMA, which is absorbed into the bloodstream through the intestinal mucosa and then converted to TMAO in the liver by flavin-containing monooxygenases (FMOs; Zeisel and Warrier, 2016; Subramaniam and Fletcher, 2018). In recent years, the relevance of TMAO to human diseases has been widely reported. Overall, elevated TMAO concentrations increase the risk of diseases such as diabetes (Tai et al., 2015), heart failure (Trøseid et al., 2015), and atherosclerosis (Dalla Via et al., 2019). TMAO has also been associated with mortality and hospitalization rates for cardiac and renal diseases such as atrial fibrillation (Tang et al., 2014), acute myocardial infarction (Suzuki et al., 2017), and chronic kidney disease (Tang et al., 2015). In addition, TMAO has been reported to be strongly correlated with the development of inflammatory bowel disease (IBD) and Alzheimer’s disease (AD; Wilson et al., 2015, 2016; Xu and Wang, 2016), and even with the outcome of immunotherapy in triple-negative breast cancer (Wang et al., 2022). However, the effect of TMAO on antibiotic efficacy is still unknown.

Recently, an interesting study has found that a high-fat diet leads to dysbiosis of intestinal flora and depletion of the microbial metabolite indole-3-acetic acid (IAA), leading to reduced antibiotic efficacy against bacterial infections (Liu et al., 2021). The study by Oliver et al. found that a diet high in fiber and low in animal protein established an association with antibiotic resistance by shaping the human gut microbiota, but the role played by the corresponding gut metabolites triggered by a specific diet needs further elucidation (Oliver et al., 2022). The intestinal microbiota carries a large number of antibiotic resistance genes (Ruppé et al., 2019; Forster et al., 2022), and those metabolites that reduce antibiotic susceptibility may exacerbate the emergence and spread of resistant bacteria. The inhibition of antibiotic efficacy by intestinal microbiota metabolites may drive the enrichment and evolution of antibiotic-resistant bacteria (Becattini et al., 2016). If so, bacterial resistance will become even more problematic. Because the influence of diet on intestinal metabolites is extensive (Koh et al., 2016; Sonnenburg and Bäckhed, 2016; Cani et al., 2019), the dietary structure of different individuals is complex. This makes it particularly important to explore the effect of intestinal flora metabolites on antibiotic efficacy.

This work explored the effect of a gut microbial-associated metabolite, TMAO, on antibiotic efficacy *in vitro*. Interestingly, TMAO reduced the susceptibility of *Escherichia coli* to a variety of antibiotics. Further data suggest that TMAO does not rely on activation of the TMAO sense-regulatory system and the classical multidrug efflux system to mediate the inhibition of antibiotic efficacy. In addition, TMAO enhanced the survival of *E. coli* under lethal urea (protein denaturant) stress and also protected *E. coli* from killing by two disinfectants (ethanol and H_2_O_2_) that cause protein damage. In conclusion, our study suggests that TMAO has the potential to improve bacterial survival under anti-biotics and other lethal stresses through its protective effect against protein denaturation/damage.

## Results

### Trimethylamine N-Oxide Increases the MIC of Antibiotics in a Concentration-Dependent Manner

**Figure 1:**
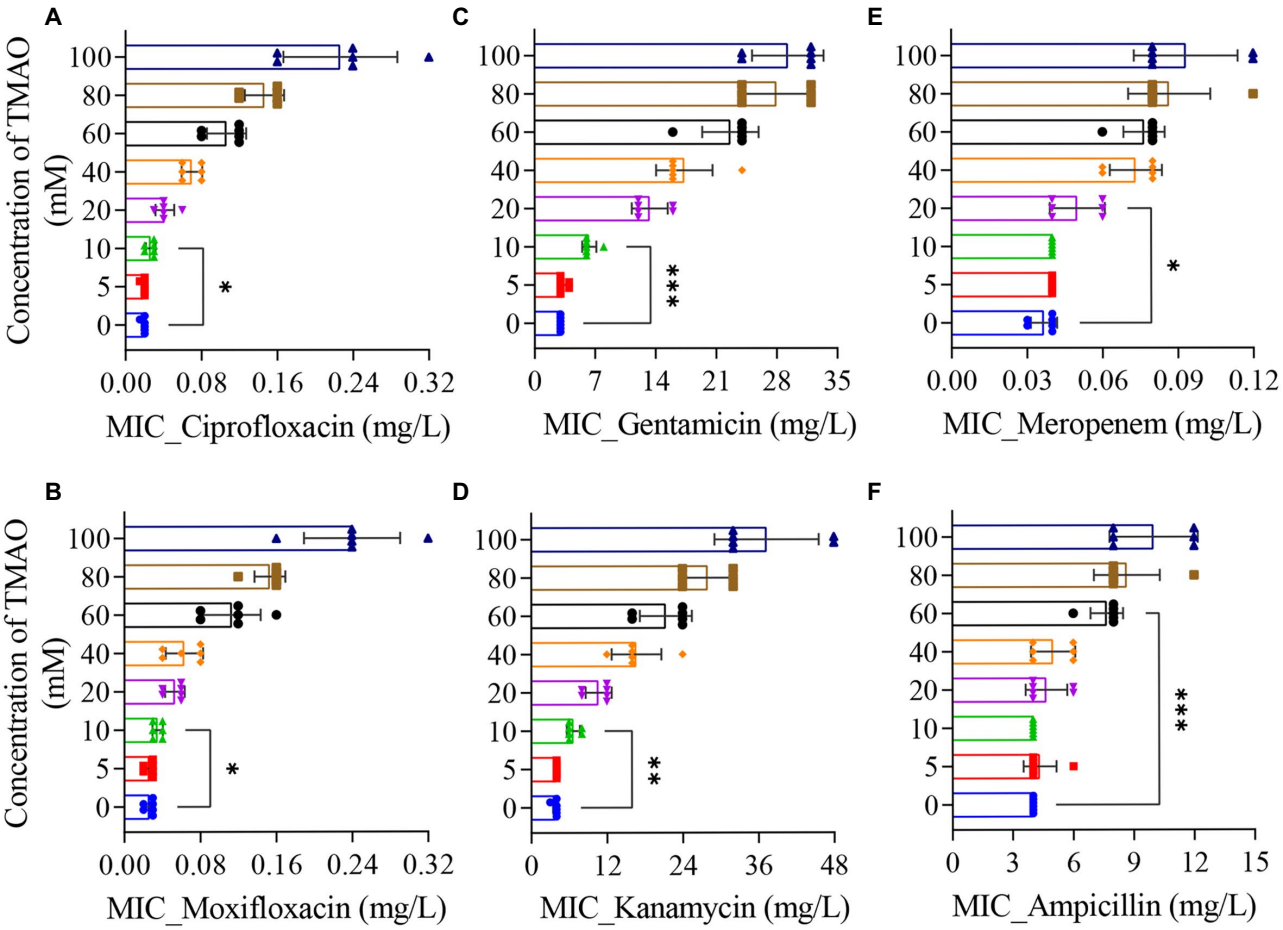
Effect of Trimethylamine N-oxide (TMAO) on minimum inhibitory concentration (MIC) of antibiotics. **(A–F)** MIC was determined by the 2-fold broth dilution method. Cultures incubated to OD_600_ = 0.2 were diluted to ~10^5^ cells/ml, mixed with various amounts of drug, and incubated at 37°C for 12 h. As needed, different concentrations of TMAO were added to the LB medium. Six individual experiments were performed. Each data plotted value represents mean ± SD. Significance determined by paired *t*-test. ^*^p < 0.05; ^**^p < 0.01; ^***^p < 0.001.

To investigate the correlation between TMAO and antibiotic efficacy *in vitro*, we added 5 ~ 100 mM TMAO to LB medium to determine the effect of different concentrations of TMAO on the minimum inhibitory concentration (MIC) of antibiotic. In the absence of TMAO, the MIC of ciprofloxacin against the wild-type strain (BW25113) was about 0.02 mg/l; under the condition of 10 mM TMAO, the MIC of ciprofloxacin was mostly 0.03 mg/l; however, when the concentration of TMAO was increased to 100 mM, the MIC of ciprofloxacin measured in six independent experiments was as high as 0.16 ~ 0.32 mg/l (Figure 1A). Similarly, TMAO also increased the MIC of moxifloxacin, another quinolone antibiotic, in a concentration-dependent manner (Figure 1B). Next, we measured the MIC of two aminoglycosides and two β-lactams under the same TMAO concentration gradient. The results showed that 10 mM TMAO more significantly increased the MIC of gentamicin and kanamycin against wild-type strain (Figures 1C,D), and the MIC promoted with increasing of TMAO concentrations. Differently, the effect of TMAO on the MIC of β-lactams required higher concentrations than that for the other two types of antibiotics. The MIC of meropenem increased to 0.06 mg/l in three out of six MIC determinations under 20 mM TMAO, compared to 0.03 mg/l (2 times)/0.04 mg/l (4 times) in the absence of TMAO. And in 40 mM TMAO conditions, the MIC of meropenem increased to 0.06 ~ 0.08 mg/l (Figure 1E). The MIC of ampicillin only increased from 4 to 6 mg/l (1 time)/8 mg/l (5 times) with 60 mM TMAO (Figure 1F). The above results suggest that TMAO increased the MIC of the three types of antibiotics against *E. coli* in a concentration-dependent manner in the range of 10 ~ 100 mM. Furthermore, by measuring the growth curve, we ruled out the possibility that TMAO affects bacterial growth and thus alters the susceptibility of *E. coli* to antibiotics (Supplementary Figure 1).

### Higher Doses of Antibiotics Are Required to Kill *Escherichia coli* Cells in the Presence of TMAO

**Figure 2:**
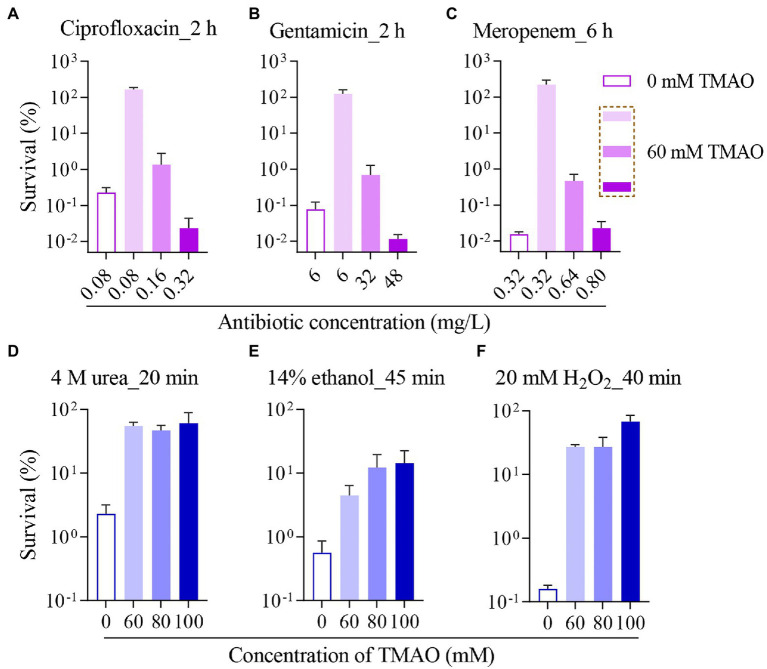
Trimethylamine N-oxide protects *Escherichia coli* from killing by antibiotics or other lethal stressors. Survival of wild-type strain incubated with 0 or 60 mM TMAO, after ciprofloxacin treatment for 2 h **(A)**, gentamicin treatment for 2 h **(B)**, or meropenem treatment for 6 h **(C)** was measured. And survival of wild-type strain incubated with different concentrations of TMAO, after treatment with 4 M urea for 20 min **(D)**, 14% ethanol treatment for 45 min **(E)**, or after 20 mM H_2_O_2_ treatment for 40 min **(F)** was measured. TMAO was added to the corresponding bacterial culture for co-incubation 30 min before antibiotic addition. Experiments were performed independently for three times. Each data plotted value represents mean ± SD.

Then, we examined the effect of TMAO on antibiotic sterilization in *E. coli*. Since ampicillin showed a significant increase in MIC under the 60 mM TMAO condition (Figure 1F), the concentration was used for subsequent experiments, and one of each of the three types of antibiotics tested was chosen as a representative. In the absence of TMAO, treatment with 0.08 mg/l (4 × MIC) ciprofloxacin (Figure 2A) or 6 mg/l (2 × MIC) gentamicin (Figure 2B) for 2 h reduced the survival of log-phase wild-type *E. coli* by approximately three orders of magnitude; treatment with 0.32 mg/l (8 × MIC) meropenem (Figure 2C) for 6 h reduced the survival of wild-type *E. coli* by about four orders of magnitude. In contrast, under 60 mM TMAO condition, neither ciprofloxacin, gentamicin, nor meropenem at equivalent absolute concentrations exhibited lethality against the wild-type strain because of the elevated MIC (Figures 2A–C, comparison of the second column with the first column). Ciprofloxacin and gentamicin demonstrated better bactericidal efficacy when sterilized with a standardized MIC (Figures 2A,B, fourth column compared to the first column). With 60 mM TMAO, meropenem required a higher multiple of MIC (10 × MIC) to achieve the bactericidal level of 8 × MIC meropenem in the absence of TMAO (Figure 2C, fourth column compared to the first column). Overall, the presence of TMAO requires higher concentrations of antibiotics for growth inhibition or bacterial killing.

### TorSTR Signaling Pathway and Typical Resistance Mechanisms Are Not Involved in the TMAO Effect on Antibiotic Sensitivity

TorSTR signaling pathway senses TMAO and activates downstream pathways in *E. coli* (Baraquet et al., 2006). TorZ (TMAO reductase), and the *torCAD* operon under positive regulation of TorR can reduce TMAO to TMA (Gon et al., 2000, 2001). To investigate the mechanism by which TMAO inhibits antibiotic susceptibility of *E. coli*, we determined the MIC of ciprofloxacin on mutant strains related to the TorSTR signaling pathway. The results showed that the MIC of ciprofloxacin against ∆*torT*, ∆*torR*, ∆*torC*, ∆*torZ*, and wild-type strains with or without 60 mM TMAO was essentially the same (Supplementary Figure 2). In addition, neither the addition of 60 mM nor 100 mM TMA changed the MIC of the above six antibiotics against the wild-type strain (Supplementary Table 1). Another TMAO-binding protein (Pietrzyk-Brzezinska and Cociurovscaia, 2022), RcdA in *E. coli*, is suggested to be involved in regulating many stress response genes (Shimada et al., 2012). The *rcdA* mutation also considerably increased the MIC of ciprofloxacin against *E. coli* (Supplementary Figure 2). This suggests that the activation of intracellular TMAO-related downstream pathways does not serve as a condition to reduce the susceptibility of *E. coli* to antibiotics.

The typical mechanisms that cause larger MIC for antibiotics include: (1) the *mar* (multiple antibiotic resistance) motif, in which MarA can alter the expression of multiple genes to confer bacterial resistance (Cohen et al., 1993; Barbosa and Levy, 2000; Duval and Lister, 2013) and (2) a more direct way of causing bacterial resistance is the efflux pump, and eight efflux pump complexes have been reported in *E. coli*, and they all contain TolC proteins (Koronakis et al., 2000; Koronakis, 2003). The results showed that deletion of the *marA* gene did not alter the MIC of ciprofloxacin or meropenem, and the increase in MIC of ciprofloxacin or meropenem against the ∆*marA* strain in the presence of 60 mM TMAO was not significantly different from that against the wild-type cells (Supplementary Figures 3A,B left). Deletion of the *tolC* gene reduced the MIC of ciprofloxacin from 0.02 to 0.0025 mg/l and that of meropenem from 0.04 to 0.0075 mg/l against *E. coli*, but TMAO significantly increased the MIC of ciprofloxacin or meropenem against the ∆*tolC* strain (Supplementary Figures 3A,B right).

### Trimethylamine N-Oxide Reduces the Lethality of Urea, Ethanol, and H_2_O_2_ Against *Escherichia coli*

After excluding the role of the above factors in the inhibition of antibiotic efficacy by TMAO, we focused on the biological activity of TMAO. It is known that TMAO is present in high concentrations in some aquatic organisms and counteracts the denaturing effects of urea and salt (Lin and Timasheff, 1994; Seibel and Walsh, 2002). Under laboratory conditions, TMAO has likewise been shown to protect protein stability under urea stress (Zou et al., 2002), and TMAO may stabilize proteins by acting as a heterogeneous surfactant for folded proteins (Liao et al., 2017). Four molar urea is commonly used as a high-intensity protein denaturant, and our experiments revealed that treated with 4 M urea for 20 min, different concentrations of TMAO (60 ~ 100 mM) showed 1 ~ 2 orders of magnitude of protection against the wild-type strain (Figure 2D), and treated with 4 M urea for 40 min, 60 mM TMAO also demonstrated significant protection, and the degree of protection by TMAO is positively correlated with the concentration of TMAO (Supplementary Figure 4A).

Further, we selected a common disinfectant, ethanol, to verify the role of the property of TMAO to stabilize proteins in protecting bacterial survival. Ethanol denatures proteins by breaking the hydrogen bonds originally present in the protein (Thomas and Dill, 1993). The data showed that 14% ethanol decreased the survival of the wild-type strain by two orders of magnitude or more than three orders of magnitude for 45 or 60 min treatment, respectively (Figure 2E; Supplementary Figure 4B), for both 45 and 60 min treatment, 60 ~ 100 mM TMAO showed protective effects. H_2_O_2_, a reactive oxide, denatures proteins through covalent modification of specific amino acid side chains, resulting in oxidative damage (Dahl et al., 2015). Multiple antibiotics have the effect of inducing protein aggregation (Cardoso et al., 2010; Tran et al., 2011; Wu et al., 2015). If TMAO can improve the survival of *E. coli* under H_2_O_2_ stress, it may also suggest that protection against protein denaturation could be a potential mechanism for TMAO to inhibit the efficacy of different antibiotics. Surprisingly, as shown in Figure 2F; Supplementary Figure 4C, 60 mM TMAO significantly increased the survival of *E. coli* under 20 mM H_2_O_2_ stress, and the protection of TMAO to bacteria against H_2_O_2_ was more concentration-dependent for 60 min of H_2_O_2_ treatment. Since TMAO is a weak oxidant, this suggests that the contribution of TMAO to bacterial survival under H_2_O_2_ is not directly by antioxidant effect.

## Discussion and Future Directions

In this work, we report the inhibitory effect of TMAO on the efficacy of various antibiotics and disinfectants. The concentration of TMAO showed a positive correlation with the MIC of quinolones, aminoglycosides, and β-lactams (Figure 1), and the lethal dose of different antibiotics against *E. coli* increased accordingly when TMAO was included (Figures 2A–C). Unexpectedly, the effect of TMAO on antibiotic MIC did not depend on downstream metabolic regulatory pathways (Supplementary Figure 2), *mar* motif, and efflux pump (Supplementary Figure 3). Similar to the direct protection of *E. coli* under urea stress (Figure 2D), TMAO also improved the survival of *E. coli* after treatment with two disinfectants, ethanol and hydrogen peroxide (Figures 2E,F).

### Protein Inactivation Is a Potentially Common Pathway for Different Stress-Mediated Bacterial Cell Death

What is the mechanism by which TMAO helps *E. coli* cope with different stresses? Understanding this question will help in the development of antimicrobial strategies and guide the development of novel antimicrobial agents or adjuvants. Quinolones act on DNA replication, aminoglycosides target ribosomal subunits, β-lactams inhibit cell wall synthesis, urea and ethanol mediate protein denaturation, and hydrogen peroxide can cause oxidative damage. Our data suggest that TMAO can reduce the stress of *E. coli* by these different stressors. One of the possible explanations is that TMAO has an unknown wide range of biological activities to modulate stress processes under different stresses, like the universal molecule metformin (Lv and Guo, 2020). The reports of TMAO in humans involved in different diseases such as cardiovascular or tumor (Janeiro et al., 2018; Yang et al., 2019; Gatarek and Kaluzna-Czaplinska, 2021) may support this opinion.

However, we prefer an alternative explanation, which is the direction of our coming studies, that TMAO may help bacteria to cope with different external stresses in a pervasive way, such as by stabilizing proteins (Krywka et al., 2008; Ganguly et al., 2020). Many conditions including temperature, osmotic pressure, ionic strength, and pH, can denature proteins (Anfinsen and Scheraga, 1975), or oxidative stress occurring from hydrogen peroxide exposure can damage proteins through covalent modification of specific amino acid side chains (Dahl et al., 2015). In terms of antibiotics, sublethal concentrations of kanamycin induce protein aggregation by reducing ribosome fidelity and causing protein misfolding (Tamás et al., 2018; Schramm et al., 2019), and these mistranslated proteins can further exacerbate aggregation by promoting the production of reactive oxygen species and lead to oxidation-sensitive protein damage (Ling et al., 2012). More importantly, several antibiotics can induce protein aggregation (Cardoso et al., 2010; Tran et al., 2011; Wu et al., 2015), and Kohanski et al. (2007) found that ROS is a common pathway for the lethality of different antibiotics and that antibiotic-induced oxidative damage is part of the lethal effects of antibiotics (Dwyer et al., 2014). Proteins are responsible for accomplishing most cellular functions and protein activity is closely related to cell death. Acute external stresses can interfere with protein quality control mechanisms, leading to extensive protein denaturation and aggregation, which further leads to the loss of protein function, impairment of critical cellular functions required for growth and survival, and ultimately cell death (Mogk et al., 2018). For example, in the recently discovered new mode of cell death, cuproptosis, lipid acylated protein aggregation, and loss of iron–sulfur cluster proteins can trigger proteotoxic stress and ultimately cell death (Tsvetkov et al., 2022). These studies on protein homeostasis and cell death strengthen our confidence in the second explanation mentioned above and suggest that problem of antibiotic resistance caused by TMAO can be alleviated by finding adjuvants that promote protein denaturation. Alternatively, other possible mechanisms in TMAO-mediated antibiotic resistance events cannot be excluded. In addition, the discovery of common pathways that can respond to different lethal stresses contributes to a better understanding of the interaction of antibiotic resistance with adverse environments, where the emergence of resistance is not only limited to the drug itself.

### Dietary-Mediated Changes in Intestinal Flora Metabolites May Influence the Effectiveness of Clinical Anti-infective Therapy

Different diets can alter the composition of the intestinal flora and the levels of microbiome-dependent intestinal metabolites (Sonnenburg et al., 2016; Frame et al., 2020; Mayneris-Perxachs et al., 2022). In particular, decreased levels of the tryptophan metabolite IAA in the intestine of mice fed a high-fat diet could antagonize the treatment of Methicillin-resistant *Staphylococcus aureus* (MRSA) or *E. coli* with several antibiotics (Liu et al., 2021). TMAO, as an intestine-derived metabolite, is formed by specific intestinal microorganisms that metabolize dietary choline and betaine into precursor TMA, which is oxidized to TMAO in the liver (Wang et al., 2011). A diverse diet rich in carnitine and choline from red meat, eggs, and shellfish (Li et al., 2018), betaine present in plants (Zeisel et al., 2003), may influence TMAO levels. Rath et al. (2021) showed that microbial flora and diet were associated with high levels of trimethylamine N-oxide in the plasma of senior individuals.

*In vitro*, our results suggest that TMAO reduces the susceptibility of *E. coli* to a variety of antibiotics in the range of 10 ~ 100 mM. After setting up the test concerning the concentration range of TMAO in normal human plasma, TMAO did not demonstrate an inhibitory effect on antibiotic efficacy (Supplementary Table 2). It might be a matter of concentration effects. Our results imply that TMAO may be a detrimental factor in anti-infective therapy. Considering the practical differences that exist between laboratory conditions and clinical situations, further clinical data are needed on what concentration of TMAO in humans interferes with antibiotic efficacy. Whether microbial flora and diet may influence anti-infective treatment efficacy by modulating TMAO levels in clinical situations also needs to be elucidated in large cohort studies that take into account individual differences and other factors. Moreover, our study and that of Liu et al. (2021) and Oliver et al. (2022) suggest that more possible intestinal metabolites should be included when testing for effects on antibiotic susceptibility and that promising protocols to counteract the inhibition of antibiotic efficacy by metabolites should be sought to inform clinical practice. If a regulated pathway of “diet-flora-metabolites-antibiotic resistance” is widespread, the control of bacterial resistance will require greater engineering.

## Data Availability Statement

The original contributions presented in the study are included in the article/Supplementary Material, further inquiries can be directed to the corresponding author.

## Author Contributions

JQ, YL, and YW performed experiments and analyzed data. JQ, YL, and Morigen conceived the experiments and wrote/revised the paper. Morigen provided experimental materials and supervised the work. All authors contributed to the article and approved the submitted version.

## Funding

This work was supported by grants from the National Natural Science Foundation of China (NSFC grant no. 32060016 to Morigen), Inner Mongolia Key Laboratory for Molecular Regulation of the Cell (no. 2021PT0002 to Morigen), and Research Program of science and technology at Universities of Inner Mongolia Autonomous Region (no. NJZZ20036 to YL).

## Conflict of Interest

The authors declare that the research was conducted in the absence of any commercial or financial relationships that could be construed as a potential conflict of interest.

## Publisher’s Note

All claims expressed in this article are solely those of the authors and do not necessarily represent those of their affiliated organizations, or those of the publisher, the editors and the reviewers. Any product that may be evaluated in this article, or claim that may be made by its manufacturer, is not guaranteed or endorsed by the publisher.
