# Supplementary material for: Trimethylamine N-Oxide Reduces the Susceptibility of Escherichia coli to Multiple Antibiotics
Source: Front Microbiol. 2022 Jul 7;13:956673. doi: 10.3389/fmicb.2022.956673 (PMC9300990; doi:10.3389/fmicb.2022.956673)
Supplement: Supplementary file 1 [file Table_1.docx]

Supplementary Material

# Supplementary Materials and Methods

**1.1** **Strains, Culture, and Reagents**

All strains used in this work were listed in Supplementary Table 3. The single mutant strains were identified using the primers listed in Supplementary Table 4. And all strains were operated under LB medium conditions at 37 ℃ with shaking at 200 rpm. Ciprofloxacin, kanamycin, ampicillin, gentamicin, urea, and ethanol were purchased from Sangon Biotech Inc. (Shanghai, China). Moxifloxacin and meropenem were purchased from Macklin Biotech Inc. (Shanghai, China). H_2_O_2_ was purchased from Sinopharm Chemical Reagent Co., Ltd. (Shanghai, China). Trimethylamine N-oxide dihydrate and trimethylamine hydrochloride were purchased from Innochem Technology Inc. (Beijing, China).

**1.2 Minimal Inhibitory Concentration (MIC) Determinations**

MIC was determined by the 2-fold broth dilution method. Cultures incubated to OD_600_=0.2 were diluted to ~10^5^ cells/mL, mixed with various amounts of drug, and incubated at 37 ℃ for 12 hours. The MIC of drugs is the concentration corresponding to the test tube with no significant visible turbidity increase relative to untreated control. Different concentrations of TMAO or TMA are added to the medium in advance as needed.

**1.3 Bacterial Survival Analysis**

Overnight grown bacterial cultures were re-cultured to OD_600_=0.2, and TMAO was added 30 minutes in advance as needed. After treatment with different antibiotics, urea, ethanol, or H_2_O_2_, the samples were serially diluted 10-fold with saline at different time points. Diluted samples (10 μL) were placed in triplicate on LB agar and cultures were incubated for 16 hours to determine the colony-forming units, relative to cultures sampled immediately after dosing, to determine the percentage survival. Because of solubility limits, 4 M of urea was dissolved directly in LB medium, filtered and de-contaminated, and pre-warmed 30 minutes in advance in a 37 ℃ incubator. Then, after the culture was grown to OD_600_=0.2, centrifuged at 8000 g for 3 min at room temperature, removed the supernatant and resuspended in an equal volume of LB medium containing 4 M urea, and the time of urea acting on bacteria was recorded immediately.

- 1. **Determination of Growth Curve**

Overnight cultures of wild-type strain were diluted 2,500-fold in fresh LB medium containing 0 mM or 100 mM TMAO, incubated at 37 ℃ with 200 rpm shaking, and then OD values were recorded continuously at 10-hour intervals.

**1.5 Statistical Analysis**

All experiments were performed independently, at least three times, as shown in the legend for each image. The graphing was done by GraphPad Prism 9 and the values plotted for each data represent the standard deviation.

# Supplementary Figures and Tables

## Supplementary Figures


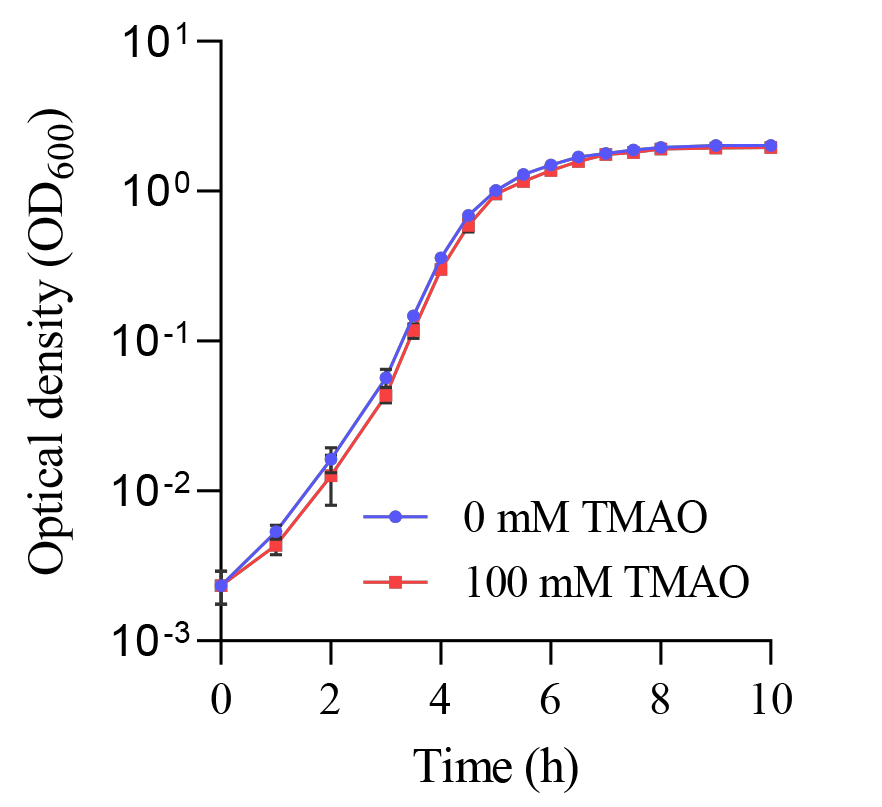


## Supplementary Figure 1. Growth curve of wild-type strain after adding 0 mM or 100 mM TMAO. Growth curve of wild-type strain after adding 0 mM or 100 mM TMAO. Data are average of three independent experiments, each data plotted value represent mean ± SD.


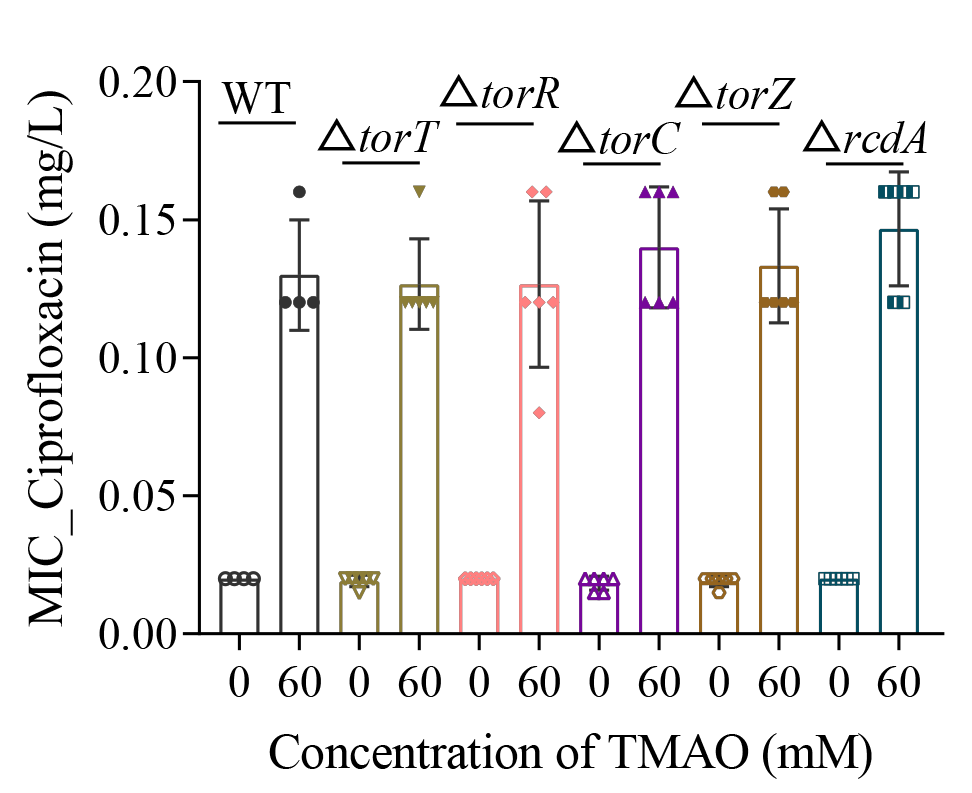


**Supplementary Figure 2.** Disruption of the TMAO regulatory pathway does not alter the effect of TMAO on ciprofloxacin MIC. MIC was determined by the 2-fold broth dilution method. Cultures containing ~10^5^ cells/mL, mixed with various amounts of drug and co-incubated at 37 ℃ for 12 hours. 0 mM or 60 mM TMAO was added to the LB medium as needed. Experiments were performed independently for four or six times. Each data plotted value represent mean ± SD.


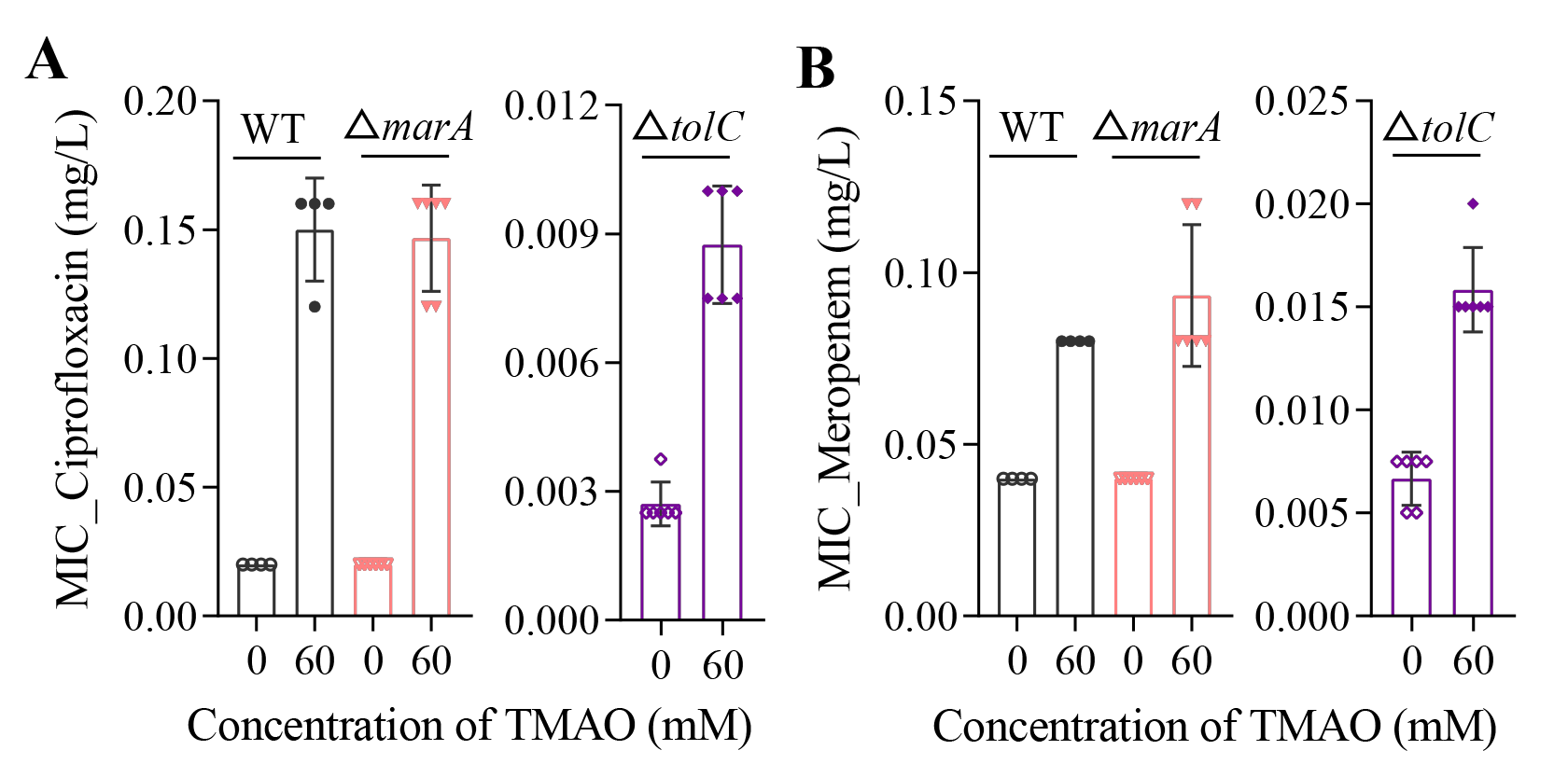


**Supplementary Figure 3.** Deficiency of *marA* or *tolC* does not change the effect of TMAO on the MIC of antibiotics. **(A, B)** MIC was determined by the 2-fold broth dilution method. Cultures containing 105 cells/mL, mixed with various amounts of drug and co-incubated at 37 ℃ for 12 hours. 0 mM or 60 mM TMAO was added to the LB medium as needed. Experiments were performed independently for four or six times. Each data plotted value represent mean ± SD.


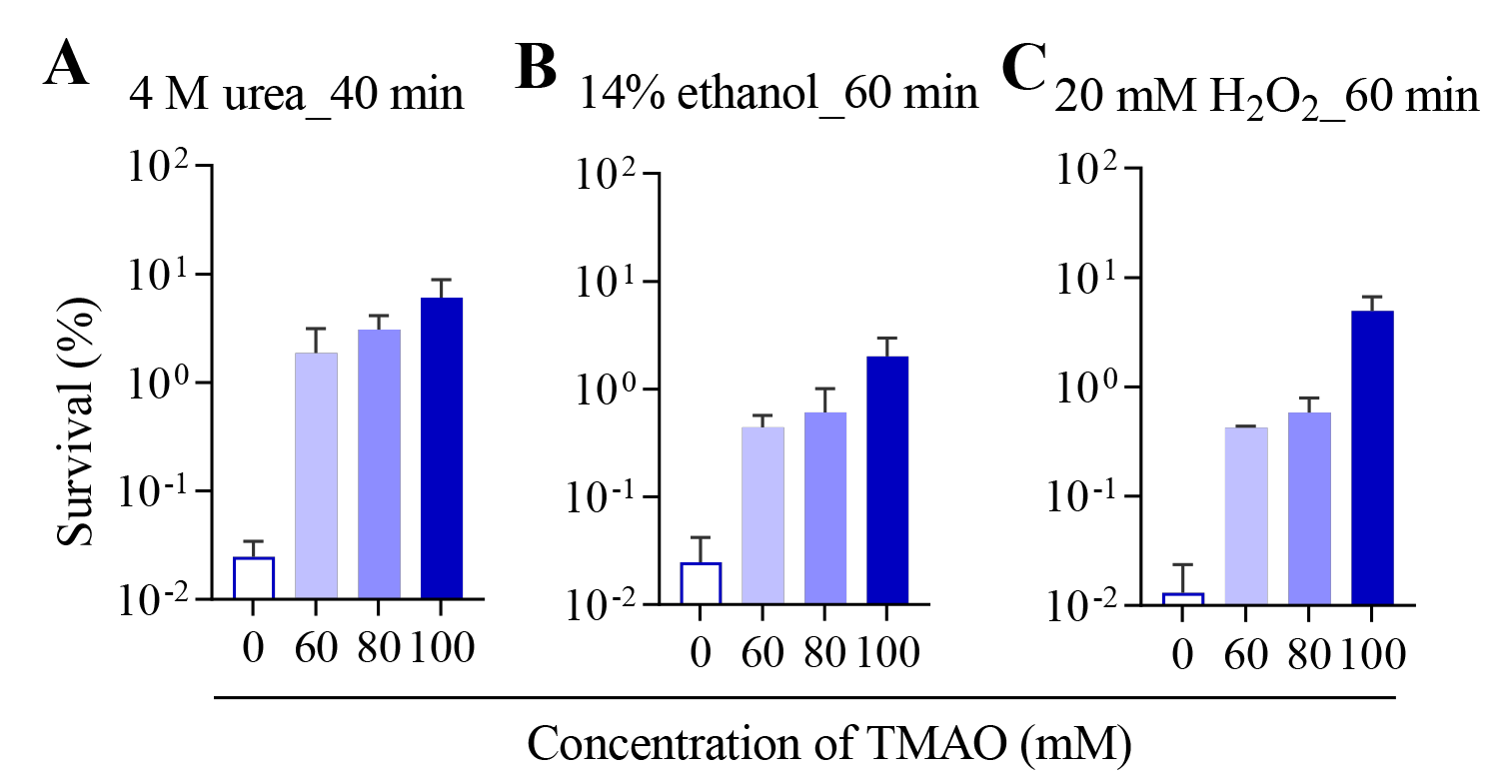


**Supplementary Figure 4.** TMAO protects *E. coli* from killing by urea, ethanol, or H_2_O_2_. Survival of wild-type strain incubated with different concentrations of TMAO, after 4 M urea treatment for 40 min **(A)**, 14% ethanol treatment for 60 min **(B)**, or after 20 mM H_2_O_2_ treatment for 60 min **(C)**. TMAO was added to the corresponding bacterial culture for co-incubation 30 min before antibiotic addition. Experiments were performed independently for three times. Each data plotted value represent mean ± SD.

## Supplementary Tables

**Supplementary Table 1.** Minimal Inhibitory Concentrations (MIC) values for different antibiotics against wild-type strain at different concentrations of TMA. (n=3)

| Concentrations of TMA  (mM) | Chemical (mg/L) | | |
| --- | --- | --- | --- |
|  | Ciprofloxacin | Moxifloxacin | Gentamicin |
| 0 | 0.02 | 0.03 | 3 |
| 60 | 0.02 | 0.03 | 3 |
| 100 | 0.02 | 0.03 | 3 |
|  | Kanamycin | Meropenem | Ampicillin |
| 0 | 4 | 0.04 | 4 |
| 60 | 4 | 0.04 | 4 |
| 100 | 4 | 0.04 | 4 |

**Supplementary Table 2.** Minimal Inhibitory Concentrations (MIC) values for different antibiotics against wild-type strain at different concentrations of TMAO. (n=3)

| Concentrations of TMAO  (μM) | Chemical (mg/L) | | |
| --- | --- | --- | --- |
|  | Ciprofloxacin | Gentamicin | Meropenem |
| 0 | 0.02 | 3 | 0.04 |
| 1 | 0.02 | 3 | 0.04 |
| 10 | 0.02 | 3 | 0.04 |
| 100 | 0.02 | 3 | 0.04 |
| 1000 | 0.02 | 3 | 0.04 |

**Supplementary Table 3.** Bacterial strains used in this work.

| Strain | Genotype | Source |
| --- | --- | --- |
| BW25113 | *E. coli* wild-type *rrnB*3 Δ*lacZ*4787 *hsdR*514 Δ(*araBAD*)567 Δ(*rhaBAD*)568 *rph-1* | (Baba et al., 2006) |
| Δ*torT* | BW25113 Δ*torT*::*neo* | (Baba et al., 2006) |
| Δ*torR* | BW25113 Δ*torR*::*neo* | (Baba et al., 2006) |
| Δ*torC* | BW25113 Δ*torC*::*neo* | (Baba et al., 2006) |
| Δ*torZ* | BW25113 Δ*torZ*::*neo* | (Baba et al., 2006) |
| Δ*rcdA* | BW25113 Δ*rcdA*::*neo* | (Baba et al., 2006) |
| Δ*marA* | BW25113 Δ*marA*::*neo* | (Baba et al., 2006) |
| Δ*tolC* | BW25113 Δ*tolC*::*neo* | (Baba et al., 2006) |

**Supplementary Table 4.** Primers used in this work.

| Primer Name | Sequence（5′--3′） | Usage |
| --- | --- | --- |
| F-*torT*-check | GGCTGATAAAGCGCAGGTTG | Identification of *torT* mutant |
| F-*torR*-check | CAGCGCAATGCCAATCACAA | Identification of *torR* mutant |
| F-*torC*-check | TAGGATTGTAATCGCGCCTGG | Identification of *torC* mutant |
| F-*torZ*-check | TCAGGCGTCTTTACAGGGTG | Identification of *torZ* mutant |
| F-*rcdA*-check | CACGCCGAGCAGAGAATACC | Identification of *rcdA* mutant |
| F-*marA*-check | TGCCAGGGCAACTAATGTG | Identification of *marA* mutant |
| F-*tolC*-check | TTTGCCAAATGTAACGGGCAG | Identification of *tolC* mutant |
| R-check^a^ | GCTTGCTGTCCATAAAACCG | Mutant identification |

F: forward; R: reverse

a: R-check is a universal downstream primer that pairs with each forward primer in the list.

R-check primer was designed inside the kanamycin resistance gene.

1. **Supplementary References**

Baba, T., Ara, T., Hasegawa, M., Takai, Y., Okumura, Y., Baba, M., et al. (2006). Construction of *Escherichia coli K-12* in-frame, single-gene knockout mutants: the Keio collection. *Mol Syst Biol* 2**,** 2006.0008. doi: 10.1038/msb4100050.
